# Extended spectrum β-lactamase producing *Enterobacteriaceae* & antibiotic co-resistance

**Published:** 2011-01

**Authors:** S.M. Rudresh, T. Nagarathnamma

**Affiliations:** Department of Microbiology Victoria Hospital Bangalore 560 002 Karnataka, India

Sir,

Extended spectrum β-lactamases (ESBLs) are plasmid mediated enzymes inactivating β-lactam antibiotics containing oxyimino group such as oxyimino-cephalosporins and oxyimino-monobactam, except cephamycins and carbapenems^1^. They are derived from the point mutation of plasmid determined TEM or SHV β-lactamases^1^,^2^. ESBLs are inhibited by clavulanic acid and placed under Bush’s functional class 2be^3^. Till date more than 200 different types of ESBLs have been described. In recent years, there is a dramatic increase in the prevalence of CTX-M type of ESBLs among clinical isolates of *Enterobacteriaceae* in Europe and Asia^4^.

ESBLs are the most evolving mechanism of antibiotic resistance among the family *Enterobacteriaceae* due to the selective pressure imposed by inappropriate use of third generation cephalosporins, most often encountered in ICU settings^2^. Plasmids coding for ESBL enzymes may carry co-resistance genes for other non-β-lactam antibiotics, thus limiting the number of useful drugs against these bacteria^5^–^7^. Reliable detection of ESBL production by clinical microbiology laboratory is essential to guide the clinicians to provide appropriate therapy. Hence this study was designed to know the presence of ESBLs among members of the family *Enterobacteriaceae* isolated at Victoria hospital, Bangalore and to know the antibiotic susceptibility pattern among ESBL producers and non-ESBL-producers.

A total of 239 consecutive, non-repetitive, clinical isolates of *Enterobacteriaceae* isolated from various clinical samples such as exudates (95), urine (71), sputum (54), blood (15) and vaginal swab (4) obtained between July 2009 and November 2009 were included in the study. Samples were processed and isolates were identified by standard laboratory methods^8^.

Antibiotic susceptibility was determined by Kirby Bauer disc diffusion method according to Clinical Laboratory Standard Institute (CLSI) guidelines^9^. Antibiotics were chosen depending on the organism and the sample and results were interpreted as sensitive or resistant as per CLSI recommendations^10^.

ESBL was confirmed by CLSI described phenotypic confirmation method along with routine antibiotic susceptibility testing^9^,^10^. A stock solution of clavulanic acid (2000 µg/ml) was prepared, aliquoted into small vials and stored at -20µC. One vial was removed just before antibiotic susceptibility testing and 5 µl of clavulunate solution was added to the cefotaxime (30 µg) disc (Hi-Media, Mumbai). A lawn of test organism was made on Mueller-Hinton agar (MHA) after adjusting the inoculum to 0.5 McFarland and cefotaxime and cefotaxime/clavulunate discs were placed along with CLSI described antibiotic discs, incubated at 37µC for 18-24 h. A zone difference of >5 mm between cefotaxime and cefotaxime/clavulunate was considered as confirmative for ESBL production.

Though CLSI described phenotypic confirmatory test is applicable for *Escherichia coli, Klebsiella pneumoniae* and *Proteus mirabilis*, an attempt was made to look for ESBL production among other members of *Enterobacteriaceae* also. Throughout the study K. pneumoniae ATCC 700603 and E. coli ATCC 25922 (HiMedia Laboratories, Mumbai) were used as positive and negative controls respectively, for ESBL production.

Among the 239 *Enterobacteriaceae* isolates, 96 (40.2%) were E. coli, 79 (33.1%) *K. pneumoniae*, 26 (10.9%) *Enterobacter* spp, 21 (8.8%) *Proteus* spp, 15 (6.3%) *Citrobacter* spp and 2 (0.8%) *Salmonella* Typhi. Prevalence of ESBL is known to vary according to geographical regions^11^,^12^. In our study, ESBL production was seen in 149 (62.3%) isolates of *Enterobacteriaceae*. Fifty four (22.6%) isolates were non-ESBL producers and 36 (15.1%) showed no zone difference in phenotypic confirmatory test. Such isolates showed uniform resistance to cefoxitin and all were susceptible to cefepime. On further testing, all the 36 isolates were found to be AmpC producers by modified three dimensional test^13^.

ESBL production was more common among isolates obtained from exudates 67/95 (70%) followed by blood 10/15 (66.7%) and urine 42/71 (59.1%). Among the 239 isolates, 136 (56.9%) were obtained from inpatients and 103 (43.1%) from out patients. ESBL production was more among the isolates from inpatients 97/136 (71.3%) when compared to outpatients 52/103 (50.5%). The AmpC producing isolates among the in- and out-patients were 22/136 (16.2%) and 14/103 (13.6%) respectively.

ESBL production was higher in *E. coli* when compared to *K. pneumoniae* and *Enterobacter* spp. AmpC β-lactamases were seen more among *Citrobacter* spp. followed by *Proteus* spp. and *E. coli* (Table I).

**Table I:** Comparison of ESBL production among clinical isolates

Organism	ESBL+ (%)	ESBL- (%)	Amp C (%)	Total
*E. coli*	65 (67.7)	15 (15.6)	16 (16.7)	96
*K. pneumoniae*	50 (63.3)	22 (27.8)	7 (8.9)	79
*Enterobacter* spp.	15 (57.7)	9 (34.6)	2 (7.7)	26
*Proteus* spp.	12 (57.1)	3 (14.3)	6 (28.6)	21
*Citrobacter* spp.	7 (46.7)	3 (20)	5 (33.3)	15
*Salmonella* Typhi	0 (00)	2 (100)	0 (00)	2
Total	149 (62.3)	54 (22.6)	36 (15.1)	239

The presence of multi-drug resistance was higher among ESBL producers and carbapenems remained the most effective drug against such isolates (Table II). Non-betalactam antibiotic susceptibility among ESBL producing organisms showed least sensitive to co-trimoxazole (23.4%) followed by ciprofloxacin (29.5%) and gentamycin (46.9%).

**Table II:** Comparison of antibiotic susceptibility pattern of ESBL, non-ESBL and AmpC producing *Enterobacteriaceae*

Drug	I	PT	NT	C	AK	G	CF	CO	CPM	CPM	NX	CB
ESBL+ (%)	100	95.3	76.5	69.1	67.7	46.9	29.5	23.4	2.6	92.8	14.2	7.1
ESBL- (%)	100	100	98.1	88.8	94.4	88.8	79.6	70.3	100	93.3	53.3	60
AmpC (%)	100	69.4	41.6	55.5	41.6	38.8	22.2	22.2	100	85.7	28.5	00

I, Imipenem (10 µg); PT, piperacillin/tazobactam (100/10 µg); NT, netilmicin (30 µg); C, chloramphinicol (30 µg); AK, amikacin (30 µg); G, gentamicin (10 µg); CF, ciprofloxacin (30 µg); CO, co-trimoxazole (1.25/23.75 µg); CPM, cefepime (30 µg); NF, nitrofurantoin (300 µg); NX, norfloxacin (10 µg); CB, carbenicillin (100 µg)

In our study freshly prepared cefotaxime/clavulunate discs were used for phenotypic confirmatory test.

The study showed ESBL producers were highly resistant to cefepime (97.3%) at standard inoculum, which is in contrast to the study of Thomson *et al*^14^ who showed inoculum effect was more for cefepime among the ESBL producing *Enterobacteriaceae*.

The present study showed 15.1 per cent of isolates were pure AmpC producers. Among the 149 ESBL producers, 50 isolates though showed zone difference of >5mm in phenotypic confirmatory test, the combination with clavulanic acid did not enhance the zone to completely susceptible levels. Such phenotype may suggest production of both ESBL and AmpC or production of multiple β-lactamases. Cefoxitin resistance in such isolates cannot be considered as indicator of AmpC production, as other mechanism of resistance such as porin channel mutation is also more often seen among ESBL producing organisms^15^. Further studies are needed for appropriate detection of combined ESBL and AmpC enzyme production among such isolates.

Occurrence of ESBL producing *Enterobacteriaceae* at our centre was higher when compared to reports from other hospitals in India^11^,^12^. The study indicated routine detection of ESBL production using phenotypic confirmatory test as simple, cost-effective and time saving method. Instead of screening and confirming ESBL production, direct phenotypic confirmatory test along with routine antibiotic susceptibility testing helped to report ESBL production within 48 h.

High degree of antibiotic co-resistance among ESBL producers emphasizes the judicious use of antimicrobials. Imipenem still remains most effective drug against ESBL producing organisms followed by piperacillin-tazobactam. The study showed phenotypic confirmatory test can reliably detect ESBL production among all the members of *Enterobacteriaceae*.
